# Experimental Infection of Swans and Geese with Highly Pathogenic Avian Influenza Virus (H5N1) of Asian Lineage

**DOI:** 10.3201/eid1401.070740

**Published:** 2008-01

**Authors:** Justin D. Brown, David E. Stallknecht, David E. Swayne

**Affiliations:** * College of Veterinary Medicine of the University of Georgia, Athens, Georgia, USA; † Southeast Poultry Research Laboratory of the US Department of Agriculture, Athens, Georgia, USA

**Keywords:** avian influenza virus, swans, geese, H5N1, highly pathogenic avian influenza, wild birds, migratory, research

## Abstract

The role of wild birds in the epidemiology of the Asian lineage highly pathogenic avian influenza (HPAI) virus subtype H5N1 epizootic and their contribution to the spread of the responsible viruses in Eurasia and Africa are unclear. To better understand the potential role of swans and geese in the epidemiology of this virus, we infected 4 species of swans and 2 species of geese with an HPAI virus of Asian lineage recovered from a whooper swan in Mongolia in 2005, A/whooper swan/Mongolia/244/2005 (H5N1). The highest mortality rates were observed in swans, and species-related differences in clinical illness and viral shedding were evident. These results suggest that the potential for HPAI (H5N1) viral shedding and the movement of infected birds may be species-dependent and can help explain observed deaths associated with HPAI (H5N1) infection in anseriforms in Eurasia.

## Abstract 2

Susceptibility to infection, duration of illness, and concentration of asymptomatic viral shedding vary between species of swans and geese.

The first indication of wild bird involvement in the Asian lineage highly pathogenic avian influenza (HPAI) virus (H5N1) epizootic occurred late in 2002 and 2003, when HPAI (H5N1) was isolated from captive and wild birds in Hong Kong Special Administrative Region, People’s Republic of China (*1*). Since these initial outbreaks, HPAI (H5N1) has continued to cause illness and death in a variety of wild birds in Asia (*2*), and in 2005 the virus was recovered from migratory waterfowl during a wild bird die-off involving primarily bar-headed geese (*Anser indicus*) at Qinghai Lake, People’s Republic of China (*3*). Although several thousand birds died in this outbreak (*4*), it is unknown how many birds, including other species, were infected and dispersed from Qinghai Lake. In the late summer through fall of 2005, Asian lineage HPAI virus (H5N1) was first detected in Europe, where it was isolated from dead wild waterfowl in several European Union member states and neighboring countries (*5*). Most of these HPAI (H5N1) isolates were recovered from a limited number of species in the order Anseriformes, including mute swans (*Cygnus olor*), whooper swans (*C. cygnus*), tufted ducks (*Aythya fuligula*), and Canada geese (*Branta canadensis*) (*5*,*6*).

Although the ability of Asian lineage HPAI (H5N1) to infect and cause death in wild birds has been documented, the epidemiology of this virus in free-ranging avian populations is unclear. Wild avian species infected by this virus in Asia have been taxonomically diverse, whereas in Europe, most deaths and HPAI virus (H5N1) isolations have been limited to only a few species of geese and swans. A growing body of genetic and epidemiologic evidence suggests that, in 2005, migratory waterfowl played a role in the geographic spread of Asian lineage HPAI virus (H5N1) to Europe (*5*,*7*). However, the virus has not been detected in clinically healthy wild birds in Europe that were not associated with ongoing bird die-offs (*8*), and no evidence has clearly shown that the virus is maintained or geographically spread by infected asymptomatic wild birds. A reliance on dead bird surveillance makes HPAI virus (H5N1) in wild waterfowl difficult to evaluate and has left several gaps in our understanding (*9*). Whether Asian lineage HPAI (H5N1) that spilled over from domestic poultry to migratory waterfowl has or can be maintained in wild avian populations or whether similar outbreaks will recur is not known.

The objective of this study was to evaluate the susceptibility and viral shedding patterns in 4 species of swans and 2 species of geese that were experimentally infected with HPAI virus (H5N1) and to then predict the ability of each species to spread the virus into new areas. Susceptibility was determined on the basis of prevalence and onset of illness and death and distribution of microscopic lesions and viral antigen. Viral shedding patterns were based on duration, route, and concentration of viral excretion. We evaluated the potential ability of a given species to geographically move the virus on the basis of the duration and viral titers associated with asymptomatic shedding.

## Methods

### Animals

We used 4 species of swans and 2 species of geese in this study: whooper swan, black swan (*C. atratus*), trumpeter swan (*C. buccinator*), mute swan, bar-headed goose, and cackling goose (*B. hutchinsii*). All birds used in this study were bred in captivity and purchased from commercial breeders in the United States. The swans were 5–6 weeks of age at the time of the experiment. This age was chosen on the basis of availability of birds and size restrictions imposed by the isolation units. Geese were ≈12 weeks of age at the time of the experiment, which corresponds to the age of juvenile waterfowl during the peak prevalence of avian influenza virus (AIV) in wild waterfowl in North America (*10*). Male and female birds were included in each species in approximately equal numbers. Infected birds for each species were housed separately in groups of 4 or 5 (inoculated and contact birds) in self-contained isolation units, which were ventilated under negative pressure with HEPA-filtered air. Sham-inoculated birds were maintained in separate units from the infected birds and grouped by individual species. The birds were maintained under continuous lighting, and food and water were provided ad libitum.

All birds used in this study were cared for in accordance with the guidelines of the Institutional Animal Care and Use Committee, as outlined in the Guide for the Care and Use of Agricultural Animals in Agricultural Research and Teaching (*11*) and under an animal use protocol approved by the Institutional Animal Care and Use Committee at the Southeast Poultry Research Laboratory (SEPRL), Agricultural Research Service (ARS), United States Department of Agriculture (USDA), Athens, Georgia, USA, and at the University of Georgia (UGA), Athens, Georgia, USA. All experiments were performed in the USDA-certified Biosafety Level 3-Ag facility at SEPRL (*12*).

### Viruses

A stock of the A/whooper swan/Mongolia/244/2005 (H5N1) (Mongolia/2005) HPAI virus was produced by second passage in 9-day-old embryonated chicken eggs. Allantoic fluid from the inoculated eggs was diluted in brain–heart infusion (BHI) medium to yield a final titer of 10^6^ median embryo infectious doses (EID_50_) per 0.1 mL (single-bird inoculum) as determined by standard procedures (*13*). A sham inoculum was prepared by diluting sterile allantoic fluid 1:100 in BHI. The Mongolia/2005 virus was originally isolated from a dead whooper swan during a large die-off of waterfowl (*14*), and the isolate was chosen for use in this study because of its known lethality in wild waterfowl under natural and experimental (*15*) conditions. In addition, this strain appears to be a genetically representative isolate from the wild bird HPAI virus (H5N1) (clade 2.2) that has been reported in Asia, Europe, and Africa (*15*). Extrapolations from our data herein were made with the assumption that the Mongolia/2005 virus is representative of the 2005–2006 Asian lineage HPAI (H5N1) isolated from dead wild birds in Eurasia.

### Experimental Design

Preinoculation serum was collected from each bird to confirm that they were serologically negative to influenza A type–specific antigens by the agar gel precipitin (AGP) test and the H5 hemagglutinin subtype by the hemagglutinin–inhibition (HI) test. In addition, oropharyngeal and cloacal swabs were collected from each bird to ensure that they were not actively infected and shedding AIV at the start of the study. Two (whooper swan and cackling goose) or 3 (trumpeter swan, black swan, mute swan, and bar-headed goose) birds from each species were inoculated intranasally (IN) with 0.1 mL of the Mongolia/2005 virus solution. After 24 hours, 2 additional birds from each species were placed in the housing unit with the inoculated birds. All birds were monitored daily for illness or death for 14 days. Illness was defined as any clinical abnormality detectable upon physical examination after inoculation with, or contact exposure to, the HPAI virus (H5N1), including weakness, cloudy eyes, respiratory difficulty, shivering, crowding, ruffled feathers, hemorrhage on the unfeathered skin, and neurologic signs, such as tremors, seizures, severe incoordination, and torticollis. Oropharyngeal and cloacal swabs were collected and then placed in BHI with antimicrobial agents (400 μg/mL gentamicin, 4,000 U/mL penicillin, and 5 μg/mL amphotericin B) from all birds at 1, 2, 3, 4, 5, 7, 10, and 14 days postexposure (dpe). At 14 dpe, blood was collected from the surviving birds for serologic testing, and the birds were euthanized with intravenous sodium pentobarbital (100 mg/kg body weight). Blood was not collected from birds that died during the course of the study. Necropsies were performed on all birds, and routine tissues were collected for histopathologic and immunohistochemical evaluation. In addition, oropharyngeal and cloacal swabs were collected from all birds that died and were stored in BHI with antimicrobial agents for virus isolation. In reporting the temporal data, 0 days postcontact (when the contact birds were placed into the cage with the inoculated birds) was assumed to be equivalent to 0 days postinoculation.

One bird from each species was inoculated IN with a sham solution and housed in a separate unit from the virus-exposed birds. Oropharyngeal and cloacal swabs and preinoculation serum samples were collected from these birds before inoculation to confirm that they were not actively infected with an AIV and were negative for serum antibodies to the type-specific A antigen and the H5 hemagglutinin. These birds were monitored for illness and death for the 14-day trial. At 14 DPE, serum was collected from each sham-inoculated bird for serologic testing, and the birds were euthanized as described above. A necropsy was performed on each bird, and samples were collected for histopathologic and immunohistochemical evaluation.

### Histopathologic Examination and Immunohistochemical Testing

Tissues samples collected at necropsy were preserved in 10% neutral buffered formalin. After fixation, the tissues were routinely processed and embedded in paraffin. Sections were cut at 5 μm and stained with hematoxylin and eosin. Duplicate sections were cut and immunohistochemically stained by using a mouse-derived monoclonal antibody (P13C11) specific for type A influenza virus nucleoprotein antigen as the primary antibody (SEPRL). The procedures used to perform the immunohistochemical testing have been previously described (*16*). Fast red was used as the substrate chromagen, and slides were counterstained with hematoxylin. Demonstration of viral antigen was based on chromagen deposition in the nucleus, with or without chromagen deposition in the cytoplasm.

### Virus Isolation

Oropharyngeal and cloacal swabs collected at necropsy were stored at –70°C until virus isolations and titrations were performed. Isolation of virus from the swabs was performed in 9- to 11-day-old embryonated chicken eggs by using standard procedures (*13*). Positive samples were also titrated in 9- to 11-day-old embryonated chicken eggs by determining the EID_50_. The minimal detectable titer from the swabs was 10^0.97^ EID_50_/mL.

### Serologic Assays

Serologic testing was performed on the pre- and postinoculation serum with the AGP and HI tests by using standard procedures (*17*). The HI tests were performed by using a 0.5% suspension of chicken erythrocytes in phosphate-buffered saline. Serum was pretreated with chicken erythrocytes to neutralize any naturally occurring serum hemagglutinins, and the first dilution on the test plate was 1:8. All HI titers >8 were considered positive.

## Results

Disease, deaths, viral distribution, and pathology differed among the swans and geese infected with the Mongolia/2005 virus strain (Table 1). Among all species of swans, 100% of infected birds died, including all birds that were directly inoculated with the virus and those that acquired the infection through contact exposure. Viral shedding was detected in each of the IN-inoculated birds (including all swan and goose species) at 1 dpe (average oropharyngeal titer at 1 day postinoculation: black swans, 10^4.30^ EID_50_/mL; mute swans, 10^3.23^ EID_50_/mL; trumpeter swans, 10^4.17^ EID_50_/mL; whooper swans, 10^4.10^ EID_50_/mL; bar-headed geese, 10^3.83^ EID_50_/mL; cackling geese, 10^3.50^ EID_50_/mL). Challenge virus was detected in oropharyngeal and cloacal swabs from every IN-inoculated and contact bird of each species studied except 1 bar-headed goose, which is described later. Viral shedding was detected in each of the contact swans by 1 dpe but was delayed in contact geese; virus was not detected until 3 dpe in the cackling geese and 2 dpe in the bar-headed geese. Similarly, there was no difference between the onset of detectable clinical signs and death in swans that were inoculated with virus and those that were exposed through contact. Contact geese, however, had a delayed onset of detectable clinical signs (cackling geese, 6.5 days; bar-headed geese, 6.5 days) and death (cackling geese, 7.5 days; bar-headed geese, 8.0 days) compared with IN-inoculated geese (clinical signs: cackling geese, 3.5 days; bar-headed geese, 3.3 days; death: cackling geese, 4.0 days; bar-headed geese, 6.0 days)

**Table 1:** Disease, death, and pathologic data from 4 species of swans and 2 species of geese exposed to highly pathogenic avian influenza virus (H5N1) by intranasal inoculation and contact with infected birds*

Species	Disease rate (d to onset)	Duration, d (range)†	Mortality rate (d to death)	Virus distribution
Black swan	5/5 (1–2)	<1 (0–1)	5/5 (2–3)	Blood vessels
Trumpeter swan	5/5 (2)	4 (3–5)	5/5 (4–6)	Brain, skin, multiple organs‡
Whooper swan	4/4 (2–4)	3 (1–5)	4/4 (4–4)	Brain, skin, multiple organs
Mute swan	5/5 (5–7)	<1 (0–1)	5/5 (5–8)	Brain, skin, multiple organs
Cackling goose	4/4 (3–7)	3 (1–9)	3/4 (4–8)	Brain, pancreas, liver, adrenal gland
Bar-headed goose	5/5 (3–7)	4 (1–8)	2/5 (6–7)	Brain

*Exposure date for each species was adjusted so that 0 d postcontact (when the contact birds were placed into the cage with the inoculated birds) was assumed to be equivalent to 0 d postinoculation. †Average duration of detectable clinical signs. ‡Adrenal gland, pancreas, liver, lungs, heart, spleen, kidneys, air sacs, trachea, intestinal parasympathetic ganglia, and gastrointestinal tract.

**Figure 1:**
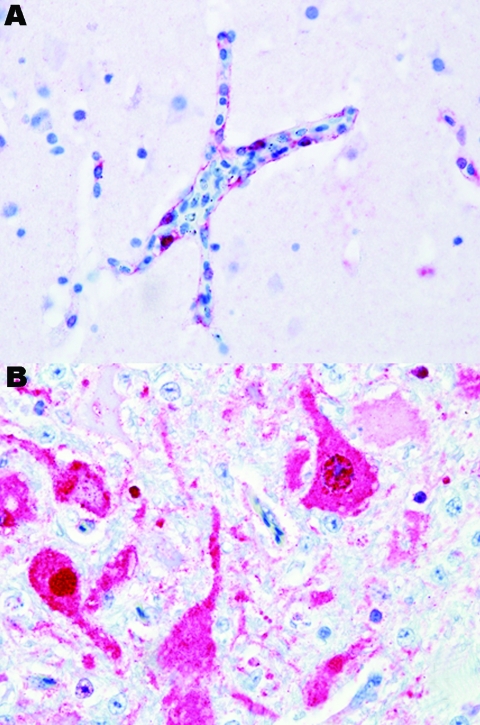
Photomicrograph of viral antigen (red). A) Endothelial cells lining a blood vessel in the brain of a black swan. B) Neurons in the brain of a mute swan. Both birds died after experimental infection with highly pathogenic avian influenza virus (H5N1). Immunohistochemical stain with hematoxylin counterstain. Magnification ×40.

Black swans were the most susceptible species examined in this study; 100% died within 2–3 dpe. Most black swans were found dead without having exhibited any clinical signs of disease. When disease was observed, it lasted for <24 hours, and clinical signs included severe listlessness and neurologic dysfunction consisting of seizures, tremors, and marked incoordination. Influenza viral antigen was detected primarily in endothelial cells lining the blood vessels throughout most visceral organs and the brain (Figure 1, panel **A**). Microscopic examination showed that all black swans that died had widespread multiorgan necrosis with mild acute inflammation, which was strongly correlated with the distribution of the virus. All of the black swans shed virus before death and, as with all birds in this study, titers were higher in respiratory secretions than in feces (Table 2). All waterfowl that died shed virus in respiratory secretions and feces; shedding generally increased with time and reached a maximum within 24–48 hours of death.

**Table 2:** Virus isolation data from 4 species of swans and 2 species of geese exposed to highly pathogenic avian influenza virus (H5N1) by intranasal inoculation and contact with infected birds*

Species	Oropharynx			Cloaca	
	Average duration†	AMT‡ (EID_50_/mL)		Average duration†	AMT‡ (EID_50_/mL)
Black swan	2 (2–3)	6.46		2 (1–2)	4.94
Trumpeter swan	5 (4–6)	6.14		4 (2–5)	3.18
Whooper swan	5 (4–6)	6.30		4 (3–5)	4.25
Mute swan	5 (3–7)	5.58		4 (3–4)	4.46
Cackling goose	5 (4–6)	5.25		3 (2–5)	3.05
Bar-headed goose	6 (5–8)	5.10		3 (0–7)	2.55§

*Exposure date for each species was adjusted so that 0 d postcontact (when the contact birds were placed into the cage with the inoculated birds) was assumed to be equivalent to 0 d postinoculation. AMT, average maximum titer; EID_50_, median embryo infectious dose. †Average duration of viral shedding in days (range). ‡AMT for birds that shed virus. All of the contact and inoculated birds shed detectable concentrations of virus by the oropharyngeal and cloacal route with 1 exception (noted below). §One bar-headed goose did not excrete detectable virus by the cloacal route, and the average maximum titer for cloacal shedding in this species was calculated based on the 4 birds with detectable cloacal shedding. If all 5 geese were included in this calculation, the average cloacal shedding would be log_10_ 2.04 EID_50_/mL.

Susceptibility was similar in the remaining 3 species of swans. Disease and death occurred later in these species, and the duration of illness, with 1 exception, was longer. Clinical signs consisted of mild to moderate listlessness, which progressively worsened to severe listlessness with neurologic signs similar to those observed with the black swans. Viral antigen was detected in the neurons (Figure 1, panel **B**), astrocytes, and other parenchymal cells of the brain and most of the examined visceral organs, as opposed to the vasculotropic distribution in black swans. Microscopic lesions were strongly associated with the anatomic location of detectable viral antigen and consisted of multifocal to coalescing necrosis with mild to moderate heterophilic inflammation. Within this category, the Mongolia/2005 virus infection in mute swans was unique. Clinical signs occurred later (5–7 dpe) in mute swans than in any of the other species examined in this study. The duration of disease in mute swans was extremely short (<24 hours) and comparable to the duration of disease in black swans. The clinical signs observed in mute swans were similar to those in the whooper and trumpeter swans. Birds in all 3 of these species shed high concentrations of virus in respiratory secretions with maximum titers approximating those of the black swans.

The 2 species of geese differed in their susceptibility to the Mongolia/2005 virus and both were less susceptible than the swan species. All of the cackling geese became sick after inoculation with the Mongolia/2005 virus, but only 3 of the 4 birds died and the remaining bird slowly recovered until clinical signs of disease were no longer apparent. The cackling geese that died exhibited severe listlessness and marked neurologic signs similar to those observed in the swans. The single goose that survived became moderately listless with ruffled feathers and cloudy eyes before clinical signs resolved but did not exhibit neurologic clinical signs during the study. This goose produced postexposure antibodies to AIV that were detected by the AGP and HI tests. Cackling geese that died had a short duration of illness (average duration 1.67 days) as opposed to the goose that survived, which exhibited detectable clinical signs for 9 days before resolution. Viral antigen in the 3 geese that died was restricted to the brain, pancreas, liver, and adrenal gland. Microscopic lesions primarily involved these organs and included multiple foci of necrosis with moderate heterophilic to lymphoplasmocytic inflammation. The single goose that survived had minimal amounts of viral antigen in the neurons of the brain and mild perivascular encephalitis. The surviving goose also shed lower concentrations of virus (maximum oropharyngeal titer 10^3.9^ EID_50_/mL) than the 3 geese that died (average maximum oropharyngeal titer 10^5.7^ EID_50_/mL), but the duration of shedding was approximately similar in both oropharyngeal and cloacal swabs.

Bar-headed geese were the least susceptible of the 6 species examined in this study. All 5 of the geese infected with the Mongolia/2005 virus exhibited clinical signs of infection; 2 of these birds died, and the remaining 3 became ill, but the clinical signs slowly resolved until they were no longer apparent. The duration of clinical signs and onset of illness and death were similar to those of the cackling geese. The bar-headed geese that died exhibited severe depression and neurologic signs. The 3 geese that survived became mildly depressed with transiently cloudy eyes but did not exhibit neurologic signs. The duration of disease was longer for the geese that survived (average duration 5.33 days) than for the geese that died (average duration 2.50 days). All 3 of these surviving geese produced antibodies to AIV that were detected by the AGP and HI tests. Viral antigen and microscopic lesions in bar-headed geese were primarily present in the brain. Viral antigen staining was more widespread in the 2 geese that died than in the 3 that survived. Microscopic lesions consisted of moderate perivascular encephalitis and neuronal necrosis in geese that died and mild perivascular encephalitis in birds that survived. The concentration and duration of viral shedding were similar between bar-headed geese that died and those that survived. Cloacal shedding was detected in all of the bar-headed geese except one, which was one of the surviving birds.

## Discussion

**Figure 2:**
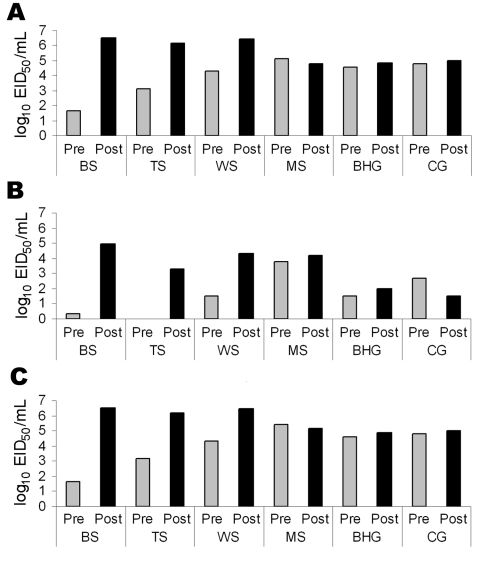
The average concentration of viral shedding in oropharyngeal (A), cloacal (B), and combined (C) routes before (pre) and after (post) the onset of clinical signs in 4 species of swans and 2 species of geese exposed to highly pathogenic avian influenza virus (H5N1) by intranasal inoculation or contact with infected birds. Viral concentrations were determined by adding viral titers before and after the onset of clinical signs for each individual bird and then using these values to calculate a pre- and postclinical average for each species. The single bar-headed goose that did not shed detectable concentrations of virus in the feces was included in the calculation of the averages for this species. EID_50_, median embryo infectious dose; BS, black swans; TS, trumpeter swans; WS, whooper swans; MS, mute swans; BHG, bar-headed geese; CG, cackling geese.

During the outbreaks of HPAI (H5N1) of Asian lineage in Europe in 2005–2006, certain duck and swan species were overrepresented in the mortality reports (*5*). Although field data from the outbreaks indicated that these waterfowl species were susceptible, their contribution to the spread of HPAI virus (H5N1) is not clear. In general, asymptomatic birds can shed virus before the onset of illness or after clinical signs have resolved. In this study, all 6 waterfowl species shed virus before the onset of clinical signs, though species-related differences were apparent (Figure 2). Some geese of both species survived infection, but none of the surviving birds actively shed detectable virus after clinical signs resolved.

Black swans were the most susceptible species to HPAI virus (H5N1) infection, with illness, deaths, and viral distribution similar to results for gallinaceous poultry (*16*). Although all of the black swans shed virus before dying, the asymptomatic viral titers were low, and the rapid course of disease would most likely preclude geographic spread of virus by this species. The high susceptibility of black swans to infection, however, would make them a good sentinel species for detection of HPAI virus (H5N1) in Australia and New Zealand, where this species is found naturally. In addition, the high concentrations of virus shed after the onset of clinical signs, but before death, would allow this species to contribute to viral transmission during a local outbreak in waterfowl.

Illness and death occurred later in whooper swans, mute swans, and trumpeter swans, which would potentially allow actively infected (and shedding) birds in these species more time to spread virus during their movements. This is particularly true for mute swans, which shed moderate to high concentrations of virus for several days without showing clinical signs of disease. The longer duration of asymptomatic viral shedding would allow this species ample time to travel and have contact with other wild birds and shared aquatic habitats. Mute swans do not migrate; however, freezing temperatures may cause many populations to move during the winter season as waterways freeze. This possibility has been suggested as a factor that contributed to the spread of HPAI virus (H5N1) in Europe during 2005–2006 (*6*).

All of the swans used in this study were inoculated at 5 to 6 weeks of age, and the high virulence observed in these species may be attributable to the young age of the birds. A negative association between age and death associated with HPAI virus (H5N1) infection occurs in ducks up to 4 weeks of age but not in chickens (*18*). Whether similar age-related differences in susceptibility to HPAI virus (H5N1) exist with swans is not known. However, the Mongolia/2005 strain was equally or more lethal for the 4 species of swans in this study as other HPAI virus (H5N1) strains have been in a variety of age-matched or younger wild avian species, including gallinaceous birds, waterfowl, and gulls (*19*,*20*). If age-related susceptibility does exist in swans, older birds may be more likely to survive the infection.

The cackling geese were more susceptible to HPAI virus (H5N1) than the bar-headed geese, as evidenced by higher proportion of deaths and more systemic viral distribution, and both of these geese species were generally less susceptible than swans. The delayed viral shedding, illness, and deaths of contact geese compared with inoculated geese provide further support that geese are less susceptible than swans. These factors may also suggest that viral transmission would occur at a slower rate within populations of these geese species than in swans. While susceptibility varied between these 2 species, both had similar onsets of disease and death, duration and concentrations of viral shedding, and duration of asymptomatic shedding. On the basis of these parameters, bar-headed geese and cackling geese would be equally efficient disseminators of HPAI virus (H5N1). Cackling geese are closely related to Canada geese, which were affected in some European outbreaks of HPAI virus (H5N1) (*6*). The lower susceptibility, relative to the other species examined in this study, of bar-headed geese was surprising considering the large number of birds that died at the Qinghai Lake outbreak in 2005. On the basis of the mortality rates in this study, many bar-headed geese may have been infected and survived; our results support the possibility that this species played a role in the transmission of HPAI virus (H5N1) to waterfowl or other wild birds outside of Qinghai Lake.

Consistent with reported wild bird mortality data from previous outbreaks, data from this study identified species-related variability in susceptibility to HPAI virus (H5N1) among wild species of waterfowl. Several important characteristics of HPAI virus (H5N1) infection differ between waterfowl species, including duration of asymptomatic shedding and duration and concentration of viral shedding. According to these characteristics, mute swans, cackling geese, and bar-headed geese may be recognized as species that pose a greater risk for transmission and spread of HPAI (H5N1). Relatively few wild avian species, rather than anseriform species as a whole, may have contributed to most of the spread of HPAI (H5N1) within Eurasia. This conclusion is consistent with observed mortality patterns during outbreaks and from the failure to detect HPAI (H5N1) in clinically normal anseriform species despite intensive sampling. This finding implies that the epidemiology of this particular lineage of AIV in waterfowl populations differs from that of low-pathogenicity AIV that naturally circulate in wild birds and that the establishment of a silent (without detectable disease and death) natural reservoir for Asian lineage HPAI virus (H5N1) strains within wild waterfowl populations may be unlikely.
